# Non-bone-derived exosomes: a new perspective on regulators of bone homeostasis

**DOI:** 10.1186/s12964-023-01431-7

**Published:** 2024-01-25

**Authors:** Ping Wang, Wenkai Shao, Zilin Li, Bo Wang, Xiao Lv, Yiyao Huang, Yong Feng

**Affiliations:** 1 Department of Orthopedics, Union Hospital, Tongji Medical College, Huazhong University of Science and Technology, Wuhan, 430022 China; 2 Department of Rehabilitation, Wuhan No. 1 Hospital, Tongji Medical College, Huazhong University of Science and Technology, Wuhan, 430030 China; 3 Department of Laboratory Medicine, Nanfang Hospital, Southern Medical University, Guangzhou, 510515 Guangdong China

**Keywords:** Exosomes, Non-bone-derived exosomes, Bone-related diseases, Bone homeostasis, Extracellular vesicles

## Abstract

Accumulating evidence indicates that exosomes help to regulate bone homeostasis. The roles of bone-derived exosomes have been well-described; however, recent studies have shown that some non-bone-derived exosomes have better bone targeting ability than bone-derived exosomes and that their performance as a drug delivery vehicle for regulating bone homeostasis may be better than that of bone-derived exosomes, and the sources of non-bone-derived exosomes are more extensive and can thus be better for clinical needs. Here, we sort non-bone-derived exosomes and describe their composition and biogenesis. Their roles and specific mechanisms in bone homeostasis and bone-related diseases are also discussed. Furthermore, we reveal obstacles to current research and future challenges in the practical application of exosomes, and we provide potential strategies for more effective application of exosomes for the regulation of bone homeostasis and the treatment of bone-related diseases.

Video Abstract

**Supplementary Information:**

The online version contains supplementary material available at 10.1186/s12964-023-01431-7.

## Introduction

The incidence of bone-related diseases is increasing, and the number of patients with bone-related diseases has increased sharply. There are approximately 250 million patients with osteoarthritis and more than 200 million patients with osteoporosis globally [1], which has brought a considerable burden to the social economy [2]. However, the existing treatment methodologies for these bone-related diseases have many problems, such as poor therapeutic effects and continued poor prognosis [3]. Intriguingly, some researchers have proposed using stem cell transplantation to treat bone-related diseases due to their differentiation potential. However, this treatment modality faces great difficulties in clinical application due to the problems the immune rejection and of high cost of the application of stem cells [4, 5]. In this context, some researchers have proposed cell-free therapy based on exosomes.

Exosomes, a kind of extracellular vesicle (EV) secreted by almost all cells, have gradually gained attention from researchers due to their crucial role in intercellular communication [6, 7]. Exosomes originate from different cellular sources through the endosomal pathway, and can transport various active substances (proteins, lipids, nucleic acids, etc.) to affect various functions of target cells [8]. Exosomes have the characteristics of high stability, nonimmunogenicity, excellent biocompatibility, and potent targeting, which overcome the limitations of traditional treatment methods [4]. Nevertheless, not all exosomes are suitable for disease treatment. Exosomes exert different specific therapeutic effects in different diseases according to the source of the parent cells. Although the role and specific mechanism of bone-derived exosomes in communication between bone-related cells have been widely demonstrated, as well as exosome extraction and preparation techniques [9–11], recent studies have shown that exosomes produced by non-bone-derived cells have better bone-targeting ability than exosomes produced by bone-derived cells. Nonbone-derived exosomes also have better effects for the treatment of bone-related diseases when used as targeted nanomedicines [12]. However, to date, there has not been a review specifically focusing on the role and mechanism of non-bone-derived exosomes in regulating bone homeostasis.

Since 2004, exosomes are the most common used term in published articles describing EVs. However, Minimal Information for Studies of Extracellular Vesicles 2014 (MISEV2014) guidelines, which were issued by the International Society for Extracellular Vesicles (ISEV) in 2014 noted that classification of exosomes and extracellular vesicles is not perfect because both terms refer to vesicles obtained by sequential centrifugation and filtration. Consequently, until a consensus is reached on the nomenclature, many researchers still recommend the use of the term EVs [13]. In 2018, ISEV published MISEV2018 to update this guideline. MISEV2018 advised that, due to the existing experimental constraints, tools affecting exosome secretion should be thoroughly assessed for involvement in the overall physiology of other EV products, non-EV products, and secreting cells. Demonstrating the specific functions of exosomes compared to other types of small EVs is still not recommended as the focus of EV studies until the effect of these tools on other factors is fully demonstrated [14]. For the aforementioned reasons, many researchers believe that using the word “small EV” in studies rather than the phrase “exosome” may be more accurate. Another large-scale study on exosomes also showed that exosomes do not carry miRNA-producing proteins, RNA-binding proteins, double-stranded DNA and histones, which we speculated to exist in exosomes in past studies [15]. Therefore, the previous research samples are likely to be mixed with non-exosome components, leading to deviations in experimental results.

Thus, in addition to gathering and analyzing articles that specifically use the term “exosome” as a keyword, this review also includes some classical research with “EV” as the key term for readers’ reference. In this review, we systematically sorted non-bone-derived exosome features, listed their known roles and mechanisms in bone homeostasis, and explained how these functions and mechanisms are connected to specific bone-related illnesses. Finally, we discussed the flaws of current research and expressed excitement about the potential clinical uses of exosomes of origins other than bone and their potential to cure bone ailments.

## Overview of exosomes

**Fig. 1:**
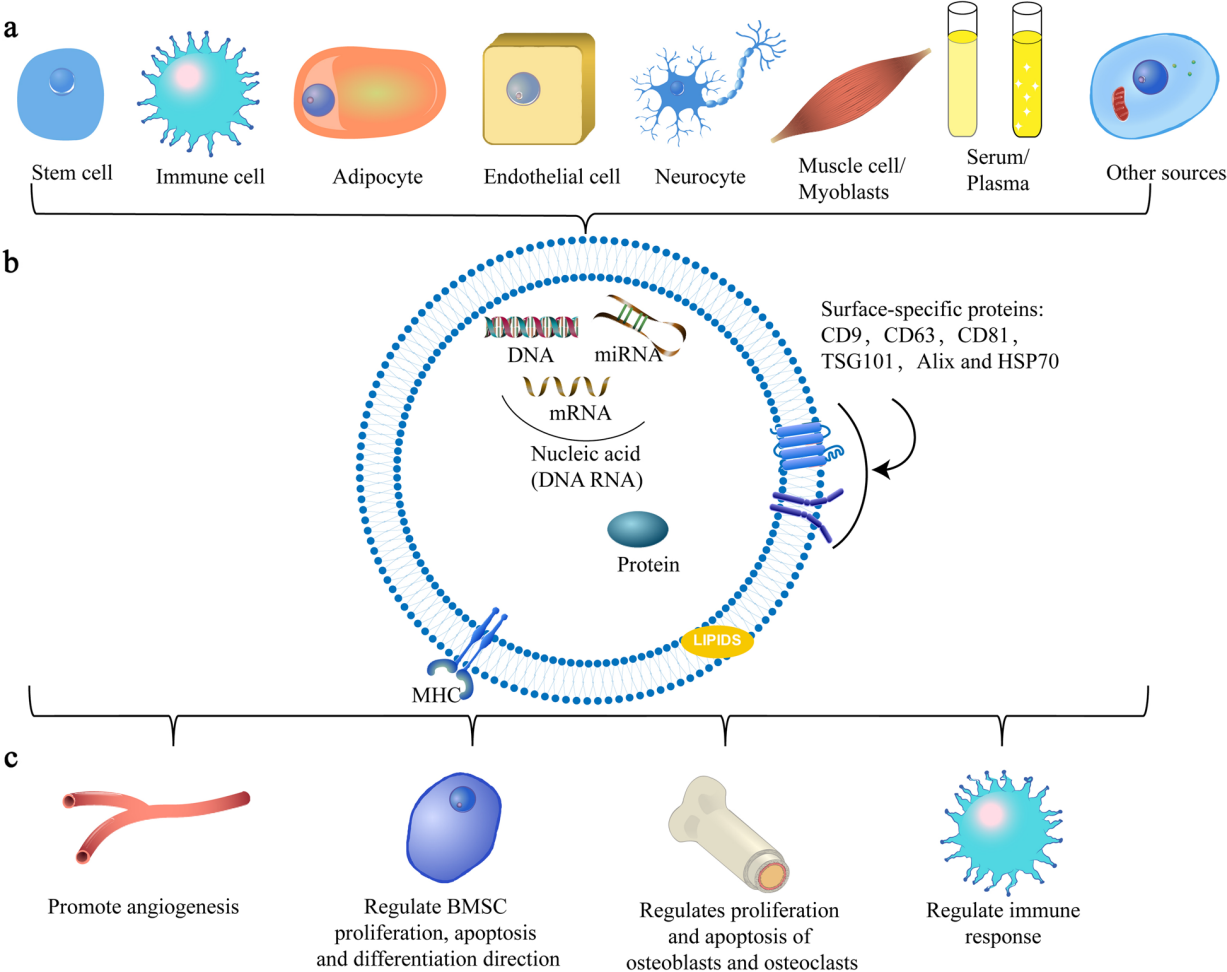
**a** The main sources of nonbone exosomes. **b** Composition of exosomes. The three main components of exosomes are lipids, proteins, and nucleic acids. Lipids are the main components of the exosome matrix, and some specific lipids and related metabolic enzymes affect the formation and release of exosomes. In addition to lipids, proteins are also enriched on the surfaces of exosomes. Various surface proteins are involved in the sorting and signal transduction of exosomes, and some proteins are also regarded as marker proteins of exosomes. In addition, there are various nucleic acid molecules, including DNA, mRNA and miRNA, that determine the specificity of exosome function. **c** The main positive effects of non-bone-derived exosomes in bone

Exosomes were first identified in in vitro experiments on sheep reticulocytes in 1983, and they were involved in the selective release of transferrin receptors during the maturation of sheep reticulocytes [16]. Exosomes are defined as extracellular vesicles between 30 and 100 nm that are secreted when the multivesicular endosome membrane and plasma membrane of eukaryotic cells fuse. These vesicles have CD63, CD81, Hsp60, Hsp70, and other distinctive protein markers [17, 18] (Fig. 1). In addition, when the exosomes were just discovered, exosomes did not receive widespread attention from researchers and were only seen as a pathway for cells to expel unwanted components. However, scientists have gradually learned that they can play important roles in metabolism, immune surveillance, angiogenesis, inflammatory responses, and tumor development by functioning as a natural carrier system to mediate intercellular signaling [6, 7].

### Composition of exosomes

The biological properties and physiological roles of exosomes are determined by their makeup, which also reflects the types of cells that produce exosomes and their conditions [7]. Lipids, proteins, and nucleic acids are the three main substances that constitute exosomes, and the types and contents of these three substances jointly determine the specificity and functions of exosomes [19].

Lipids are one of the most important components of exosomes, and specific lipids and related metabolic enzymes affect the formation and release of exosomes. Exosomes have greater lipid content than their parent cells, including cholesterol, sphingomyelin (SM), phosphatidylserine (PS) and glycosphingolipids, which are present in concentrations approximately 2–3 times higher than those of the cells themselves [20]. Lipids mainly exist in exosome membranes, and phosphatidyl-ethanolamine (PE), phosphatidylinositol (PI), PS, phosphatidic acid (PA), phosphatidylcholine (PC), ceramide, cholesterol, sphingomyelin, glycosphingolipids and some other lower-abundance lipids make up the exosome membrane under the glycan canopy on the outermost surface of the exosome [20, 21].

There are a variety of proteins in exosomes that affect their sorting and signal transduction processes [22]. Whatever the origin of the exosome, almost all exosomes include proteins involved in membrane transport and fusion (such as RAB GTPases, annexins, and flotillin), proteins that create multivesicular bodies (MVBs) (such as Alix and TSG101), tetraspanins (such as CD9, CD63, and CD81) and heat-shock proteins (such as HSP60 and HSP90) [8, 17, 23]. Among these, Alix and CD63 are enriched in some exosomes and are considered marker proteins of exosomes.

The nucleic acids in exosomes are the primary mediators of their functions. Exosomes contain single-stranded and double-stranded DNA, as well as coding and noncoding RNA [24–26]. After exosomes bind to target cells, their RNA is transferred into the cells and alters the gene expression of the cells, thereby affecting the state of the human body [24, 27–29]. Among these RNAs, miRNAs and mRNAs are currently the most widely studied and play a crucial role in the functioning of exosomes. miRNAs, can be transported by exosomes to target cells, where they can regulate specific gene expression and biological functions [26], and the mRNAs carried by exosomes can be translated after entering another cell [24]. DNA in exosomes has received far less attention than RNA. Only a few studies have demonstrated that exosomes can minimize apoptosis caused by the DNA damage response by eliminating damaging cytoplasmic DNA [30]. Moreover, some DNA carried by exosome may be employed as an early detection marker of cancer [25, 31].

At present, Vesiclepedia [32], ExoCarta [33] and EVpedia [34] are the three relatively complete exosome-related databases. These three databases have sorted the composition, physiological characteristics, sources, and molecular properties of exosomes and provide a series of tools to assist exosome research that are helpful for researchers who conduct in-depth research on exosomes.

### Biogenesis of exosomes

**Fig. 2:**
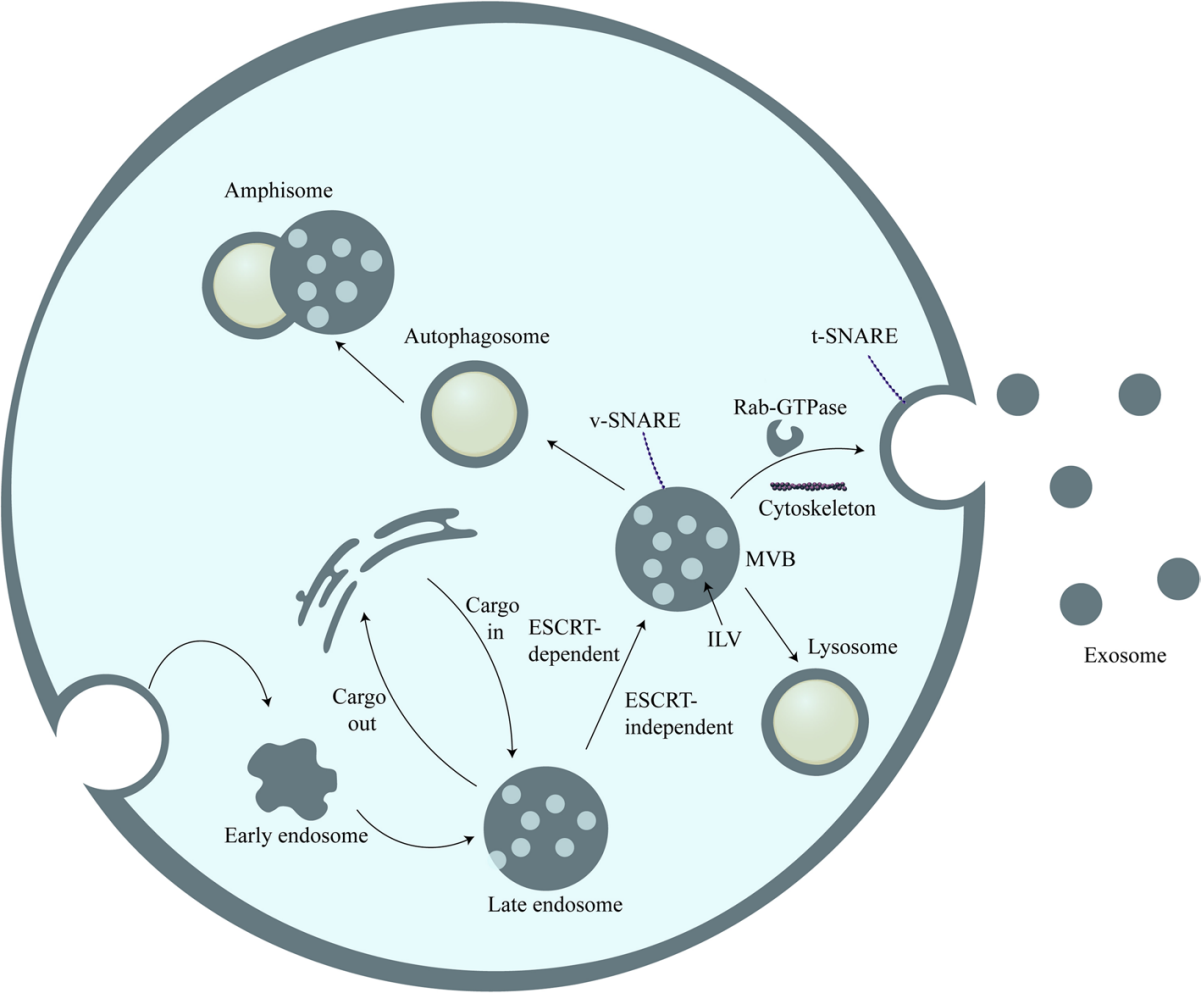
Biogenesis and secretion of exosomes. (I) Endocytosis occurs on the surface of the cell membrane and buds inwards to form early endosomes. (II) Early endosomes mature into late endosomes. (III) Through ESCRT-dependent or ESCRT-independent pathways, endosomes achieve cargo sorting and ILV biogenesis and finally form MVBs. (IV) Most MVBs are degraded in the cell after fusion with lysosomes, and some fuse with autophagosomes to form amphisomes. (V) Under the regulation of Rab GTPase, the SNARE complex, the cytoskeleton, molecular motors and other substances, a small proportion of MVBs fuse with the plasma membrane and release their ILVs to the outside of the cell in the form of exosomes

Exosome biogenesis starts with endocytosis at the cell membrane surface, and early endosomes are formed by inwards budding (Fig. 2). Intraluminal vesicles (ILVs) gradually form as early endosomes mature, the endosomal membrane invaginates [35], and then the cells sort proteins, lipids, and nucleic acids into endosomes through specific mechanisms. During this process, early endosomes mature into late endosomes and multivesicular bodies (MVBs), but only a few MVBs eventually fuse with the plasma membrane and result in secretion of exosomes, while most MVBs eventually fuse with lysosomes and are degraded inside the cell. With the help of proteins found on their surfaces such as CD63, a small proportion of MVBs fuse with the plasma membrane to release exosomes into the extracellular environment [6, 11, 17, 36]. Some studies now also suggest that MVBs can also fuse with autophagosomes and generate amphisomes, which may fuse with the plasma membrane and secrete exosomes with other cargo [37–39].

A complex called the “endosomal sorting complex required for transport” (ESCRT) plays a key role in exosome biogenesis and comprises approximately 30 single proteins, including ESCRT-0, ESCRT-I, ESCRT-II, ESCRT-III and some specific proteins such as VPS4, VTA1 and ALIX [40, 41]. ESCRT is mainly involved in endosomal sorting and ILV biogenesis during exosome biogenesis [42–44]. During this process, cargo marked by lysine-63-linked polyubiquitin chains is recognized and captured by the ubiquitin-binding site on ESCRT-0 and is then delivered to ESCRT-I [44–48]. Subsequently, ESCRT-I promotes the invagination of the endosomal membrane with the joint action of ESCRT-II [49, 50] and achieves cleavage of the ILV neck with the joint actions of ESCRT-III and VSP4 [44, 51] to cause the cargo to enter the endosome. Through continuous activation of this pathway, the sorting of endosomes and the biogenesis of ILVs are achieved.

Although ESCRT plays a major role in the generation of exosomes, some studies have shown that the biogenesis of exosomes also relies on a pathway independent of ESCRT. ILV production still occurs even if ESCRT is either ineffective or destroyed [52, 53]. This ESCRT-independent pathway relies on ceramide production by neutral sphingomyelinase 2 (nSMase2) [54, 55].

### Exosome secretion

Exosomes carried by MVBs are secreted after the fusion of MVBs with the plasma membrane [56], and molecular switches (small GTPases), the cytoskeleton (microfilaments and microtubules), molecular motors (dynein and kinesin) and the membrane fusion apparatus (SNARE complex) play an important role in this process [57]. Among these factors, the most important is the Rab GTPase, which interacts with the cytoskeleton and is crucial for vesicle budding, transport, placement, and fusion with the membrane [58, 59]. The cytoskeleton and molecular motors are involved in the trafficking of MVBs, while the fusion of MVBs to the membrane is mediated by the SNARE complex [60]. The SNARE complex, composed of proteins on the budding vesicle membrane (v-SNAREs) and on the cell membrane (t-SNAREs), is activated by the cooperative action of Rab11 and intracellular calcium [61]. After the SNARE complex is activated, v-SNAREs and t-SNAREs can fit vesicles and cell membranes together, and then the two membranes fuse to mediate the release of exosomes by vesicle exocytosis and ultimately achieve the release of exosomes [62].

## The effects on bone by bone-derived exosomes

Bone-derived exosomes mainly refer to those produced by osteoblasts, osteoclasts, osteocytes and BMSCs. The role of these bone-derived exosomes in bone has been fully investigated, the specific mechanism of their action has been demonstrated in vivo and in vitro (Table 1), and relevant clinical trials have been carried out successively.

**Table 1:** Effects of bone-derived exosomes on bone

Cell Source	Exosome Cargo	Cargo Function	Role in Bone	Reference
BMSC	–	Activate the BMP-2/Smad1/RUNX2 and the HIF-1α/VEGF signaling pathways.	Enhances osteogenesis, angiogenesis and bone healing.	[63]
BMSC	–	Promote the expression of related proteins in the MAPK signaling pathway.	Promotes osteoblast proliferation.	[64]
BMSC	miR-146a-5p, miR-503-5p, miR-483-3p, miR-129-5p	Activate the PI3K/Akt and MAPK signaling pathways	Promotes osteogenic differentiation of BMSCs.	[65]
BMSC	miR-122-5p	Downregulate expression of SPRY2 through RTK/Ras/MAPK signaling pathway.	Promote the proliferation and differentiation of osteoblasts.	[66]
BMSC	miR-206	Increase expression of OCN and BMP, decreased expression of Elf3	Promote the proliferation and differentiation of osteoblasts.	[67]
BMSC	miR-19b	Repress the expression of WWP1 or Smurf2 and elevates KLF5 expression through the Wnt/β-catenin signaling pathway.	Promote the differentiation of human BMSCs into osteoblasts, increase bone formation and reduce bone resorption.	[68]
BMSC	miR-146a	Decreased expression of Smad4 and NF2 proteins.	Promote angiogenesis.	[69]
BMSC	miR-218	Downregulate the expression of Wnt signaling pathway inhibitors (SOST, DKK2, SFRP2).	Promote commitment and differentiation of BMSC	[70]
BMSC	miR-128-3p	Inhibite the expression of Smad5.	Inhibition of osteogenic differentiation of BMSCs.	[71]
BMSC	miR-31a-5p	Inhibit the expression of SATB2, E2F2 and RhoA	Promote adipose differentiation and aging of BMSCs, inhibit osteogenic differentiation of BMSCs, and promote osteoclastogenesis.	[72]
Osteoblast	miR-503-3p	Inhibit the expression of Hpse.	Inhibit osteoclast differentiation.	[73]
Osteoblast	RANKL	Binding to RANK of osteoclast precursors.	Promotes the formation of osteoclasts.	[74]
Osteoblast	–	Decreased expression and activity of the ALP gene.	Promotes the catabolism of BMSCs.	[75]
Osteoclast	miR-214	Inhibit the expression of ATF4.	Inhibits the activity of osteoblasts.	[76]
Osteoclast	miR-214-3p	Inhibit mRNA levels of osteoblast activity-related marker genes.	Inhibit the activity of osteoblasts and reduce bone formation.	[77]
Osteocytes	miR-181b-5p	Activate the PTEN/AKT and BMP2/Runx2 signaling pathways.	Promotes the proliferation and osteogenic differentiation of HPDLSC.	[78]
Osteocytes	miR-218	Inhibit the expression of SOST.	Promotes osteogenic differentiation of BMSCs.	[79]

Exosomes derived from BMSCs (BMSC-Exos) exhibit biological functions similar to those of BMSCs and are the most critical exosomes affecting osteogenesis and promote the formation of osteoblasts and chondrocytes through different intracellular pathways. For example, the BMP-2/Smad1/RUNX2, HIF-1α/VEGF, MAPK and PI3K/Akt signaling pathways can all be activated by BMSC-Exos to promote osteogenesis and angiogenesis [63–65]. The impact of BMSC-Exos on bone is mainly achieved through the miRNAs carried by these exosomes (such as miR-122-5p [66], miR-206 [67], miR-19b [68], miR-146a [69], miR-218 [70], miR-328a-5p, miR-31a-5p [80], miR-199b [81] and miR-196a [82]), which can promote bone formation through various pathways. However, not all BMSC-Exos have a positive effect on osteogenesis. Studies have demonstrated that the expression of miR-31a-5p, which promotes osteoclastogenesis and bone resorption, is increased in BMSC-Exos from aged rats [72]. miR-128-3p in exosomes extracted from BMSCs of aged rats was also shown to inhibit bone healing by inhibiting the expression of Smad5 [71]. Therefore, although BMSC-Exos can promote osteogenesis in most cases, under the influence of age, pathology or other internal and external factors, they may in turn promote osteoclastogenesis and bone resorption, so particular attention should be given to the impact of these factors when studying BMSC-Exos.

Osteoblasts and osteoclasts cooperate to maintain bone homeostasis, and their exosomes are essential to this process [2, 74, 83]. Exosomes have been demonstrated to regulate two types of cells by transferring genetic data under certain physiological circumstances [84]. Qing et al. confirmed that osteoblast-derived exosomes downregulated the expression of the heparanase gene through miR-503-3p, thereby inhibiting the differentiation of osteoclasts [73]. It has also been demonstrated that osteoblast-derived exosomes carry essential transcription factors related to osteogenesis, which can encourage the osteogenic differentiation of BMSCs [85, 86]. In contrast, miR-214 in osteoclast-derived exosomes has been demonstrated to suppress osteoblast activity and cause bone loss [76] . This study also showed that exosomes can be secreted into the blood, and their presence in circulation may help to identify bone loss. miR-214-3p secreted by osteoclasts showed a similar effect in experiments with aging ovariectomized (OVX) mice [77]. However, some studies have also shown that exosomes released by some osteoblasts carry RANKL protein, which can promote the formation of osteoclasts through the RANKL/RANK signaling pathway [74]. Meanwhile, exosomes secreted by osteoclasts carrying RANK protein exosomes prevent this process by competitively binding RANKL. This is therefore another process requiring mutual coordination between osteoblasts and osteoclasts. Under some pathological conditions, exosomes from osteoblasts may exhibit different effects than usual; for example, exosomes derived from osteoblasts from patients with hip osteoarthritis and osteoporosis were shown to lead to enhanced catabolism in BMSCs [75]. This demonstrates that to avoid unexpected results when studying and employing exosomes obtained from osteoblasts and osteoclasts, it is important to consider the pathophysiological state of the body and the cells that supply them.

Osteocytes are the main cells that constitute bone, and the various secretions they produce play a key role in bone homeostasis, although there are few studies in this area at present. Existing studies have demonstrated that mechanical strain-induced osteocyte-derived exosomes can promote osteogenic differentiation of human periodontal ligament stem cells (HPDLSCs) through BMP2/Runx2 [78]. In addition to mechanical stress, the function of osteocyte-derived exosomes is affected by cytokines secreted by muscle, and it has been shown that miR-218 is inhibited in myostatin-treated exosomes. Such miR-218-inhibited exosomes reduce the osteogenic differentiation of BMSCs in vivo. After the addition of exogenous miR-218, this inhibition vanished, indicating the potential of miR-218 carried by osteocyte-derived exosomes in the treatment of bone disorders [79].

In summary, bone-derived exosomes are closely linked to the mechanisms of bone disorders and play a significant role in the regulation of bone homeostasis. Although there are still many unanswered questions about particular clinical uses, numerous studies have shown that they have promising futures. Before they can be used clinically, more clinical trials are needed to prove their safety and efficacy, and suitable bioengineered materials need to be designed to cooperate with them.

## The effects on bone of non-bone-derived exosomes

Nonbone-derived exosomes can be divided into exosomes derived from nonosteocytes in the bone microenvironment and other non-bone-derived exosomes. The former mainly originate in immune cells, fat cells and endothelial cells, which exist in the bone microenvironment. The latter includes exosomes originating in most types of stem cells except BMSCs, as well as exosomes from various sources, such as nerve cells and muscles. These exosomes have impacts on bone resorption, bone formation, and the development of many pathological bone processes. In the following sections, we will first describe nonosteocyte exosomes that reside in the bone microenvironment, and then we will discuss those exosomes of nonbone origin outside bone tissue. We will also discuss issues within the current research and provide an outlook on their potential clinical applications.

## Exosomes derived from nonbone tissue cells in the bone marrow microenvironment

### Effects on bone metabolism of exosomes derived from immune cells

#### Exosomes derived from macrophages

As an important effector of the immune system, macrophages affect immune defense and inflammation. Macrophages polarize into M1 and M2 types in the body. Proinflammatory M1 macrophages produce inflammatory factors that damage tissues, while anti-inflammatory M2 macrophages suppress inflammation and remove damaged tissue fragments. The cooperation of the two promotes bone healing [87]. Cellular communication between macrophages and mesenchymal stem cells affects bone homeostasis, and different types of macrophages (M0, M1, and M2) have different effects on bone homeostasis [87–91] (Table 2).

**Table 2:** Effects of macrophages-derived exosomes on bone

Cell Source	Exosome Cargo	Cargo Function	Role in Bone Metabolism	Reference
M2 macrophage	miR-378a	Increase the expression of MSC osteoinductive gene BMP2	Promotes osteogenic differentiation of BMSCs	[87]
M2 macrophage	miR-690	Activate IRS-1/TAZ signaling pathway	Promotes osteogenic differentiation of BMSCs and inhibits adipogenic differentiation of BMSCs.	[92]
M2 macrophage	miR-5106	Inhibits the expression of SIK2 and SIK3	Promotes osteogenic differentiation of BMSCs.	[93]
M2 macrophage	miR-26a-5p	Promote the expression of ALP, RUNX-2, OPN, and Col-2	Promotes osteogenic differentiation of BMSCs and inhibits adipogenic differentiation of BMSCs.	[94]
M2 macrophage	IL-10 mRNA	Promotes the expression of IL-10 and activates the IL-10/IL-10R pathway.	Promote osteogenesis, inhibit osteoclastogenesis.	[95]
M2 macrophage	Multiple differentially expressed microRNAs (DE-miRNA)	Upregulated the expression of ALP and OCN	Promote the osteogenic differentiation of PDLSCs	[96]
M1 macrophage	miR-155	Inhibit the expression of BMP2, BMP9 and RUNX2	Reduced osteogenic differentiation.	[87]
M1 macrophage	miR-98	Downregulates the expression of DUSP1 and activates the JNK signaling pathway.	Increased osteoclastogenesis, exacerbate bone loss in patients with osteoporosis.	[97]

Exosomes derived from M2 macrophages (M2-Exos) can inhibit adipogenesis and promote osteogenic differentiation of BMSCs, which is mediated by the miR-690/IRS-1/TAZ signaling pathway [92]. With intervention by M2-Exos, the expression levels of miR-690, IRS-1 and TAZ in the signaling pathway increased, thus enhancing the effect of the pathway, in which TAZ drives osteogenic differentiation and inhibits adipogenic differentiation by interacting with PPARγ and Runx2 [98], while IRS-1 is able to increase the expression of TAZ [99]. In addition, M2-Exos can also target and inhibit the expression of salt-inducible kinase 2 and 3 (SIK2 and SIK3) [93] or promote the expression of multiple transcription factors, proteins, or hormones in the body, such as RUNX-2, Col-292, ALP, OCN and IL-10 to promote osteogenic differentiation [94–96]. Due to the functions of M2-Exos in promoting bone formation, researchers have combined stromal cell-derived factor-1α (SDF-1α) and M2 macrophage-derived exosomes (M2D-Exos) with a hyaluronic acid (HA)-based hydrogel precursor solution to synthesize an injectable hydrogel. This hydrogel promotes the migration and proliferation of BMSCs and human umbilical vein endothelial cells (HUVECs), thereby promoting osteogenesis and angiogenesis in vivo and in vitro, which is beneficial for promoting fracture healing [100].

M1 macrophage-derived exosomes (M1-Exos) exacerbate bone loss and osteoporosis. Compared with M2 macrophages which promote osteogenesis, there are few studies on the mechanism by which M1 macrophages exacerbate bone loss. Current research shows that miR-155 in M1-Exos can inhibit the expression of BMP2, BMP9 and RUNX2 [87]; miR-98 can downregulate DUSP1 and activate the JNK signaling pathway [97]; and miR-222 can inhibit the expression of the antiapoptotic gene Bcl-2 [101]. Interestingly, although M1 and M2 macrophages typically have opposing effects on bone, a recent study found that LOC103691165 levels in M1 and M2 macrophages in the fracture microenvironment were comparable, and both M1 and M2 macrophages can secrete exosomes containing LOC103691165, which promotes BMSC osteogenesis [102].

In addition, researchers discovered that EVs derived from M2 macrophages can transport macrophage reprogramming factors and proteins responsible for M2 macrophage generation and migration. In response to the actions of the factors and proteins carried by these M2 macrophage-derived EVs, M1 macrophages found in inflamed synovial joints of rheumatoid arthritis patients are rapidly polarized towards the M2 phenotype. This phenomenon leads to a reduction in joint swelling, synovial inflammation, bone erosion, and articular cartilage damage in patients with rheumatoid joint disease [103]. It was discovered that combining macrophages with intrafibrillar mineralized collagen (IMC) biomaterials can lead to the promotion of MSC osteogenesis through the BMP2/Smad5 pathway. This study highlights the potential clinical use of extracellular vesicles derived from macrophages in combination with biomaterials for the treatment of bone-related diseases [104].

In conclusion, the polarization of macrophages can lead to changes in the types and quantities of miRNAs in exosomes secreted by them, which results in M1-Exos and M2-Exos having almost completely different regulatory effects on bone homeostasis. In most cases, this change causes M1 exosomes to inhibit osteogenesis, while M2 exosomes tend to prevent lipogenesis and promote osteogenesis. This may be a potential therapeutic target for bone-related diseases. By modulating the polarization of macrophages or by directly using exosomes derived from macrophages, researchers can develop many new strategies to address complex clinical needs.

#### Exosomes derived from other immune cells

In recent years, the use of exosomes originating from immune-related cells other than macrophages for bone-related applications has steadily become more common. Previous studies have demonstrated the beneficial effects of dendritic cell-derived exosomes on bone metabolism in vivo. Dendritic cell-derived exosomes selectively release cytokines (such as IL-10 and TGFβ1) during inflammation, which can promote the recruitment of regulatory T-cells, leading to the inhibition of bone resorptive cytokines and a reduction in osteoclastic bone loss [105]. In addition, through miR-335, mature dendritic cell-derived exosomes can also induce BMSCs to differentiate into osteoblasts [106]. Previous studies have also demonstrated that monocytes can promote osteogenic differentiation by increasing the expression of RUNX2 and BMP-2 in BMSCs [107]. Additionally, in patients with gout, the expression of miR-1246 is significantly increased in exosomes secreted by monosodium urate (MSU)-stimulated neutrophils, which elevates the expression of RANKL in osteoblasts and decreases the production of ALP and OPG, decreasing the viability of osteoblasts [108].

Overall, in the regulation of bone homeostasis and the treatment of bone-related diseases, the exosomes released by other immune cells are not as clinically valuable as those derived from macrophages. However, exosomes released by other immune cells can also directly affect bone homeostasis through immune regulation, thereby regulating the development of bone-related diseases. This modulatory effect may be positive or negative, and the specific circumstances and how to apply these findings need further study by researchers.

### Effects on bone by adipocyte-derived exosomes

Adipocytes are considered endocrine organs that secrete numerous hormones and cytokines to help regulate metabolism in the body, and hormones such as estrogen, leptin, and adiponectin secreted by adipocytes are crucial for bone homeostasis.

Some scientists have examined how adipose-derived exosomes (AD-Exos) affect bone homeostasis. Pan et al. showed that miR-34a carried by AD-Exos inhibited M2 polarization of macrophages by inhibiting Krüppel-like factor 4 (Klf4) [109]. Similarly, studies by Zhang et al. also showed that miR-1224 in AD-Exos inhibits macrophage M2 polarization and promotes the release of inflammatory factors by blocking the Wnt/β-catenin pathway upon entering bone marrow-derived macrophages (BMDMs) [110]. Through these effects, AD-Exos inhibit osteogenic differentiation under normal conditions, which is not conducive to bone formation in the body. Interestingly, in the Ti particle-induced osteolysis model, miR-34a carried by AD-Exos can target NLRP3 in macrophages and inhibit its expression, thereby preventing the M1 polarization of macrophages, which resulted in the alleviation of osteolysis induced by Ti particles after bone prosthesis replacement [111].

In conclusion, the functions of AD-Exos and the specific mechanisms affecting bone homeostasis vary depending on different pathophysiological conditions. Further research is required to investigate its role in bone homeostasis under different physiological and pathological models and ascertain its clinical value.

### Effects on bone by endothelial cell-derived exosomes

Endothelial cells (ECs) are highly active cells in the inner layer of blood vessel walls that can secrete a variety of substances that are vital to the body [112]. Some researchers have noted that exosomes derived from endothelial cells (EC-Exos) have stronger bone targeting than exosomes derived from osteoblasts and BMSCs and are more likely to be internalized by BMDMs. Once internalized, exosomes can inhibit osteoclast activity by upregulating miR-155 to inhibit osteoporosis [12].

A recent study showed that steroid-induced avascular necrosis of the femoral head (SANFH) could be modulated by VEGF-modified vascular endothelial cell (VEC)-derived exosomes (Exos) (VEGF-VEC-Exos). When VEGF-VEC-Exos reach BMSCs, VEGF activates the MAPK/ERK signaling pathway, which in turn inhibits adipogenic differentiation and speeds up bone production in SANFH rats [113]. And it has been demonstrated that NEAT1-carrying exosomes from HUVECs dramatically improve macrophage M2 polarization and encourage osteogenesis with the assistance of alginate/gelatin methacrylate (GelMA) interpenetrating polymer network (IPN) hydrogels [114]. Additionally, some researchers have obtained PD-L1-enriched EC-Exos through genetic engineering, and these EC-Exos can specifically bind PD-1 on the surfaces of T-cells to prevent their activation and induce MSCs to differentiate into osteogenic cells. Embedding these EC-Exos into an injectable hydrogel and injecting it into the fracture site has been shown to suppress early hyperactive inflammation and promote fracture healing without affecting the functions of distant organs such as the spleen [115].

Recent studies have demonstrated that exosomes derived from endothelial cells exhibit a higher potential for therapeutic utility and a greater ability to target bone than exosomes derived from osteoblasts and BMSCs. The utilization of EC-Exos in conjunction with bioengineering technology has yielded numerous positive results, significantly augmenting its function in maintaining bone homeostasis and treating bone disorders, these findings suggest that EC-Exos could play a crucial role in treating bone-related disorders and regulating bone homeostasis, making them a promising area of focus in exosome research.

## Exosomes derived from nonbone tissue cells outside the bone marrow microenvironment

### The effect of exosomes derived from stem cells

#### Exosomes derived from umbilical cord-derived mesenchymal stem cells (ucMSC-exos)

Umbilical cord-derived mesenchymal stem cells (uc-MSCs) can secrete exosomes that play roles in various biological or pathological processes. Comprising a diameter of approximately 100 nm and expressing CD9, CD63 and CD81 [116], ucMSC-Exos have been shown to be therapeutic agents against various diseases [117]. The proteomic analysis of ucMSC-Exos showed that there were 431 unique proteins in ucMSC-Exos. These proteins were mainly involved in biological processes such as leukocyte activation and collagen metabolism induced by the immune response and were significantly enriched in the cell adhesion molecule binding process [118].

While ucMSC-Exos have a limited capacity for osteogenic differentiation [119], their main contribution to bone production is to stimulate angiogenesis [116] (Table 3). The results of in vitro experiments demonstrate that ucMSC-Exos can stimulate endothelial progenitor cells (EPCs) to proliferate, migrate, and differentiate into angiogenic cells. The Wnt4/β-catenin pathway is an essential pathway by which exosomes stimulate angiogenesis [120]. Additionally, ucMSC-Exos can dramatically promote angiogenesis and fracture healing by increasing the expression of hypoxia-inducible factor-1α (HIF-1α) and vascular endothelial growth factor (VEGF) [116]. Mechanistic studies have shown that exosomes can also upregulate the NOTCH1/DLL4 pathway through miR-21 [121], inhibit the expression of SOX5 and EZH2 through miR-21-5p [122], and target SPRED1 and activate the Ras/Erk signaling pathway through miR-126 [123], thereby promoting vascular formation and proliferation and ultimately promoting bone formation.

**Table 3:** Effects of umbilical cord-derived mesenchymal stem cell-derived exosomes on bone

Cell Source	Exosome Cargo	Cargo Function	Role in Bone Metabolism	Reference
ucMSC	Wht4	Activate the Wnt/β-catenin signaling pathway in endothelial cells.	Promote angiogenesis.	[120]
ucMSC	–	Promotes the expression of VEGF and HIF-1α.	Promote angiogenesis and enhanced fracture repair.	[116]
ucMSC	–	Promotes the expression of VEGF and BMP2.	Promote angiogenesis and osteogenesis.	[124]
ucMSC	miR-21	Upregulate the NOTCH/DLL4 signaling pathway.	Promote angiogenesis.	[121]
ucMSC	miR-21-5p	Suppresses the expression of SOX5 and EZH2.	Promote angiogenesis and osteogenesis	[122]
ucMSC	miR-126	Activate the SPRED1/Ras/Erk signaling pathway.	Promote angiogenesis, proliferation and migration.	[123]
ucMSC	–	Promote the expression of BMSCs anabolic-related indicators and inhibit the expression of catabolic-related indicators.	Promote the proliferation of BMSCs and inhibit their apoptosis.	[125]
ucMSC	–	Activate the AKT signaling pathway.	Promotes BMSC proliferation and osteogenesis.	[126]
ucMSC	–	Promote the expression of Wnt3a,β-catenin.	Promotes osteogenesis and fracture healing.	[127]
ucMSC	miR-365a-5p	Activate the Hippo signaling pathway.	Promote osteogenesis and preventing the development of GIONFH.	[128]

In addition to the promotion of angiogenesis, an experiment in which ucMSC-Exos were directly added to autologous bone marrow stem cells (ABMSCs) showed that these exosomes promoted anabolism and inhibited catabolism in ABMSCs, hence promoting cell proliferation and preventing apoptosis [125]. ucMSC-Exos can also directly promote osteogenic differentiation by stimulating the AKT signaling pathway [126], or by promoting the expression of β-catenin and Wnt3a [127]. In a glucocorticoid (GC)-induced osteonecrosis of the femoral head (GIONFH) disease model, animal experiments confirmed that miR-365a-5p in ucMSC-Exos can effectively promote osteogenesis and prevent the deterioration of GIONFH by activating the Hippo signaling pathway [128]. In terms of practical application, Wang L. et al. synthesized a coralline hydroxyapatite (CHA)/silk fibroin (SF)/glycol chitosan (GCS)/difunctionalized polyethylene glycol (DF-PEG) self-healing hydrogel (CHA/SF/GCS/DF-PEG hydrogel) as a stable carrier of ucMSC-Exos for the treatment of bone defects [129].

In addition to previously identified exosomes, other studies have found that extracellular vesicles secreted by mesenchymal stem cells derived from neonatal umbilical cords (ucMSC-EVs) contain antiaging signals. These ucMSC-EVs can restore the progressive functional decline of tissues due to aging and enhance bone formation by transferring proliferating cell nuclear antigen (PCNA) into adult bone marrow mesenchymal stem cells (AB-MSCs) [130]. Moreover, another study showed that ucMSC-EVs can inhibit the PTEN expression level of target cells and increase the expression of AKT by transferring miR-23a-3p (carried by ucMSC-EVs), thereby promoting cartilage regeneration. Biomaterials designed based on these EVs have also demonstrated active results in in vivo experiments [131].

The umbilical cord is a cost-effective source of MSCs, and ucMSC-Exos have high therapeutic relevance due to their ability to significantly increase angiogenesis while having limited osteogenic function. Research related to ucMSC-Exos will help us to develop cell-free therapies for bone-related diseases based on the capabilities of the exosomes, researchers have tried to apply it to the treatment of osteoarthritis, and have obtained gratifying results [132]. In this context, one of the current targets in the field of exosome research should be the study of bioengineering technologies to achieve exosome enrichment at target sites in vivo and targeted therapy. This will help researchers better utilize the regulatory effect of ucMSC-Exo on bone homeostasis and apply it clinically.

#### Exosomes derived from adipose tissue-derived mesenchymal stem cells (AMSC-exos)

Lee et al. showed that AMSCs can stably produce exosomes [133]. The secretory functions of adipose MSC-derived exosomes (AMSC-Exos) are the strongest among BMSC-Exos, AMSC-Exos, and ucMSC-Exos, and proteomic analysis of AMSC-Exos revealed 457 types of proteins present in AMSC-Exos. These proteins are mainly involved in the activation of white blood cells as a result of the immune response [118].

Numerous studies conducted in the early years of this line of research established the significance of AMSC-Exos in immunological disorders, oxidative stress, and inflammation [133, 134], as well as their impact on bone homeostasis [135] (Table 4). For example, AMSC-Exos can inhibit the activation of the NLRP3 inflammasome in osteoclasts and reduce the apoptosis and bone resorption of osteoblasts to alleviate osteoporosis [136–138]. Moreover, AMSC-Exos regulate osteogenesis by regulating the polarization of macrophages [139], and miR-451a in AMSC-Exos can target migration inhibitory factor (MIF) and inhibit its expression, thereby promoting macrophage M2 polarization and ultimately promoting bone healing [140].

**Table 4:** Effects of adipose-derived mesenchymal stem cell-derived exosomes on bone

Cell Source	Exosome Cargo	Cargo Function	Role in Bone Metabolism	Reference
AMSC	–	Promotes the expression of Bcl-2 and inhibits the expression of Bax.	Inhibits osteocyte apoptosis and osteoclastogenesis.	[136]
AMSC	–	–	Attenuate TNF-α-induced osteoblast apoptosis.	[137]
AMSC	–	Suppress NLRP3 inflammasome activation in osteoclasts	Reduce bone resorption.	[138]
AMSC	miR-451a	Reduce the expression of MIF.	Promote macrophage M1-to-M2 polarization.	[140]
AMSC	miR-130a-3p	Inhibit the expression of SIRT7 and increase the expression of proteins related to Wnt signaling pathway.	Promote osteogenic differentiation of ADSCs	[141]
AMSC	–	Inhibit the expression of Nrf2.	Attenuates apoptosis in Dex-induced GIOP model and alleviates GIOP development.	[142]
AMSC	–	Activate the Wnt/β-catenin signaling pathway to enhances the osteogenic potential of BMSCs.	Enhance the ability of bone repair and regeneration in vivo, promote fracture healing.	[143]

In addition to promoting osteogenesis by affecting immune regulation, oxidative stress and inflammatory processes, AMSC-Exos can also promote bone formation in other ways. Previous studies have demonstrated that the osteogenic effect of AMSC-Exos is closely related to the Wnt signaling pathway. For example, miR-130a-3p in AMSC-Exos promotes osteogenic differentiation by downregulating the expression of SIRT7 and upregulating the expression of Wnt signaling pathway-related proteins [141]. Pretreatment of AMSC-Exos with TNF-α can enhance the expression of Wnt-3a in exosomes and further improve their ability to promote osteogenic differentiation [144]. AMSC-Exos also antagonize hypoxia and serum deprivation (H/SD)-induced apoptosis by promoting the radio of Bcl-2 and Bax, and AMSC-Exos can reduce osteoclastogenesis by inhibiting the expression of RANKL [136]. Furthermore, AMSC-Exos have been demonstrated to attenuate dexamethasone-induced apoptosis and oxidative stress by promoting the expression of Nrf2 in osteoblasts, inhibiting the development of glucocorticoid-induced osteoporosis (GIOP) [142]. The ability of AMSC-Exos to increase the osteogenic potential of BMSCs by activating the Wnt3a/β-catenin signaling pathway has also been demonstrated in a rat model of nonunion fracture healing, which offers an innovative idea for the treatment of fracture nonunion during diabetes and other diseases [143].

Adipose-derived extracellular vesicles (AMSC-EVs) and exosomes (AMSC-Exos) have been shown by numerous studies to exhibit superior osteogenesis compared to EVs from other sources [145], and these studies have further shown that AMSC-EVs have a stronger ability to promote the proliferation and differentiation of BMSCs compared with BMSC-EVs and synovial mesenchymal stem cell-derived extracellular vesicles (SMSC-EVs) [146]. Their applications have been limited, however, because of their poor homing and retention. Some researchers have therefore combined AMSC-EVs with the representative pentapeptide (among fibrin-binding peptides) cysteine–arginine–glutamic acid–lysine–alanine (CREKA) to form CREKA-functionalized AMSC-EVs (CREKA-AMSC-EVs). CREKA-AMSC-EVs not only have the osteogenic effects of AMSC-EVs themselves, but can also accurately target fibrin to accumulate and be retained in areas of bone with defects [147]. Li et al. immobilized AMSC-Exos on polydopamine-coated PLGA (PLGA/pDA) scaffolds, and in vivo and in vitro experiments showed that exosomes were slowly released from the scaffold at a relatively fixed speed and enhanced bone regeneration [148]. Another group of researchers showed similar effects in promoting osteogenesis and bone defect repair by combining AMSC-Exos with gelatin sponge/polydopamine scaffold (GS-PDA-Exos) [149]. These studies have greatly improved the prospect of the clinical use of these exosomes.

In conclusion, AMSC-Exos have been shown to interact well with biomaterials. AMSC-Exos have better osteogenic properties than BMSC-Exos and are one of the most promising means of improving the effectiveness of biomaterials for cartilage and bone regeneration. But as with all sources of exosomes, they have several drawbacks that limit their use, such as low production technology yields and the difficulty of using exosomes alone to achieve complete tissue regeneration in serious environments such as large bone defects. However, bioengineering technology may be able to compensate for these drawbacks, for example, some researchers have proposed to collect exosome mimics (EM) from MSCs to improve the yield and its ability to regulate tissue repair [150]. As these drawbacks are addressed one by one, AMSC-Exos may become a superior treatment method for our clinical treatment of bone-related diseases.

#### Exosomes derived from endothelial progenitor cells (EPC-exos)

EPC-Exos are spherical or cup-shaped vesicles with diameters of 50–150 nm that induce osteogenic differentiation of BMSCs [151]. The stimulating effects of EPC-Exos on osteogenesis are mainly achieved by promoting angiogenesis (Table 5). EPC-Exos can not only stimulate the proliferation, migration and angiogenesis of endothelial cells by downregulating SPRED-1 in a miR-126-dependent manner to promote bone formation [152], but can also upregulate the functions of endothelial cells by increasing the expression of angiogenesis-related genes such as VEGF-A and VEGFR-2 [153], thereby promoting osteogenesis by promoting angiogenesis.

**Table 5:** Effects of endothelial progenitor cells and other stem cells-derived exosomes on bone

Cell Source	Exosome Cargo	Cargo Function	Role in Bone Metabolism	Reference
EPC	miR-126	Downregulate the expression of SPEED1 and activate the Raf/ERK signaling pathway.	Enhances proliferation, migration and angiogenesis of endothelial cells.	[152]
EPC	–	Promote the expression of angiogenesis-related molecules such as VEGF-A and VEGFR-2.	Enhance proliferation, migration and angiogenic potential of endothelial cells.	[153]
EPC	LncRNA-MALAT1	Inhibits the activity of miR-124.	Promote the recruitment and differentiation of osteoclast precursors in the early stage of bone repair and promote bone repair.	[154]
EPC	–	Upregulate GPX4, systemX^c−^ and cysteine levels while reducing MDA and ROS production	Inhibits programmed osteoblast death.	[155]
EPC	miR-126	Upregulate the expressions of proteins of Erk1/2 and Bcl-2	Enhance proliferation and migration of osteoblasts, reduce apoptosis.	[156]
hiPSC	–	Enhance ALP activity, up-regulate the mRNA and protein expression of osteogenesis-related genes in BMSCs.	Promote the proliferation and osteogenic differentiation of BMSCs, promote angiogenesis.	[157]
hiPSC	–	Activation of PI3K/Akt signaling pathway in endothelial cells.	Promote angiogenesis.	[158]
SMSC	–	–	Enhanced proliferation and anti-apoptotic activity of BMSCs.	[159]
IPFP MSC	–	–	Promote macrophage M2 polarization, accelerate tendon-bone healing and intra-articular graft remodeling after ACLR.	[160]
hDPSC	–	Increased expression of osteogenic genes such as RUNT2, ALP, OCN.	Promotes osteogenic differentiation of BMSCs.	[161]
PSC	surface-associated tetraspanins	–	Induce proliferation, migration and osteogenic differentiation of osteoprogenitor cells	[162]
hESC	miR-302c	Downregulate the expression of NLRP3	Inhibite H_2_O_2_-mediated pyroptosis of nucleus pulposus cells	[163]

During the process of bone repair, early osteoclast formation is needed in addition to the need for EPC-Exos to stimulate new blood vessels. Studies have shown that EPC-Exos contain a high level of lncRNA-MALAT1, which can interact with miR-124 in bone marrow-derived macrophages (BMDMs) and inhibit their activity, thereby enhancing the recruitment and differentiation of osteoclast precursors to promote bone repair [154, 164]. EPC-Exos also inhibit glucocorticoid-induced osteoporosis by inhibiting the ferroptotic signaling pathway in osteoblasts [155] and can promote the rate of proliferation and reduce the rate of apoptosis of osteoblasts through the miR-126-mediated ERK1/2/BCl-2 pathway, thus promoting bone regeneration [156].

Overall, EPC-Exos can not only stimulate angiogenesis, which in turn stimulates osteogenesis, but also prevent osteoporosis by inhibiting the ferritin pathway of osteoblasts and stimulating the recruitment and differentiation of osteoclast precursors during the process of bone repair. Diverse functions of EPC-Exos may satisfy some complicated clinical demands in the future and play a crucial part in the treatment of some complex orthopedic illnesses, provided that some of the frequent problems they confront prior to clinical use are resolved.

#### Exosomes derived from other stem cells

An increasing number of exosomes derived from different types of stem cells have been studied by researchers. There are additional types of stem cell-derived exosomes that have been demonstrated to affect bone homeostasis in addition to those derived from the three types of stem cells listed above [2]. Stem cells primarily stimulate BMSC proliferation, angiogenesis, and the creation of an osteoinductive environment to promote osteogenesis [5] (Table 5). Exosomes secreted by mesenchymal stem cells derived from human-induced pluripotent stem cells (hiPSC-MSC-Exos) have a dose-dependent effect on BMSC osteogenesis in vivo [157], and can promote angiogenesis and osteogenesis by activating the PI3K/Akt signaling pathway [158]. Based on this principle, Zhang et al. combined hiPSC-MSC-Exos with β-tricalcium phosphate (β-TCP) and designed a hiPSC-MSC-Exo/β-TCP composite scaffold for repairing bone defects [165].

Experiments in a rat model of glucocorticoid (GC)-induced osteonecrosis of the femoral head (ONFH) showed that synovial-derived mesenchymal stem cell-derived exosomes (SMSC-Exos) can enhance the proliferation and antiapoptotic capabilities of BMSCs [159]. Infrapatellar fat pad mesenchymal stromal cell-derived exosomes (IPFP-MSC-Exos) have been shown to enhance osteogenesis by promoting the M2 polarization of macrophages [160]. In addition to their effects on macrophage polarization, IPFP-MSC-Exos have been shown to alleviate osteoarthritis (OA) by inhibiting apoptosis, promoting matrix production, and reducing the expression of catabolic factors in vivo. IPFP-MSC-Exos carry miR-100-5p, which can inhibit the mTOR autophagy pathway to maintain cartilage balance and protect articular cartilage from damage in OA mice. Currently, IPFP-MSC-Exos may be isolated from human IPFPs clinically collected from OA patients undergoing arthroscopic surgery, which is a practical and feasible method of exosome acquisition. In the future, IPFP-MSC-Exos could be an effective treatment for OA [166]. In addition, exosomes derived from human dental pulp stem cells (hDPSCs) have osteogenic effects comparable to BMP-2 in vitro [161], and tissue engineering scaffolds that are designed based on hDPSCs have also been demonstrated to accelerate bone healing in vivo [167]. Human perivascular stem cell-derived exosomes, relying on their surface-associated tetraspanins, can also promote osteogenesis [162].

Exosomes derived from human embryonic stem cells (hESC-Exos) have been shown to prevent H_2_O_2_-mediated pyroptosis of nucleus pulposus cells by downregulating the NLRP3 inflammasome during intervertebral disc degeneration (IVDD), and miR-302c carried by hESC-Exos plays an important role in this process [163]. Studies on extracellular vesicles derived from hESCs (hESC-EVs) have also demonstrated the potential of these exosomes in preventing age-related bone loss by activating classical signaling pathways such as Wnt, AMPK, and PTEN. hESC-EVs promote the expression of genes related to antiaging, cell proliferation, and osteogenic differentiation in BMSCs [168]. Moreover, some researchers have produced and isolated circRNA3503-loaded EVs from SMSCS (circRNA3503-OE-EVs). Both in vivo and in vitro experiments have shown that circRNA3503-OE-sEVs can prevent the progression of OA by relieving inflammation-induced apoptosis, improving the imbalance between ECM synthesis and ECM degradation, promoting chondrocyte renewal and alleviating the progressive loss of chondrocytes [169].

Exosomes derived from various types of stem cells can affect bone homeostasis in a variety of ways, allowing for the use of exosomes rather than stem cells for the treatment of damaged tissues or bone-related diseases, thereby avoiding some adverse effects such as the immune rejection seen with the direct use of stem cells. Exosomes have good therapeutic prospects for bone-related diseases, and further research is needed to address their current shortcomings for clinical applications.

### Effects on bone by nerve-derived exosomes

The relationship between the nervous system and bones has always been a hot spot that has attracted extensive attention from researchers, our past studies have also demonstrated the regulatory effect of nerves on bones [170, 171]. There is an interesting clinical phenomenon: traumatic brain injury (TBI), one of the most severe types of brain damage, can accelerate bone production, meaning that TBI patients typically have a higher speed of bone recovery [172, 173]. Numerous scientists have researched this unique clinical phenomenon, but none have been able to provide a convincing justification. In the past few years, the connection between bones and nerves was gradually discovered due to the expansion of exosome research, and the mechanism of this special clinical phenomenon has also been reasonably explained.

A study by Bai et al. demonstrated that after TBI, exosomes released from injured neurons can specifically target bone via FN1 and inhibit the expression of FOXO4 and CBL proteins to promote osteogenesis; miR-328a-3p and miR-150-5p carried by exosomes are crucial in this process [174]. Yang et al. also examined this issue and found that after TBI, nerve-derived exosomes express higher levels of osteoblast-associated miRNAs [175]. In addition, another recent study also showed that accelerated bone formation after TBI may be related to the formation of a sympathetic nerve-mediated local anti-inflammatory environment in the bone marrow [176].

Additionally, it has been demonstrated that exosomes produced by Schwann cells (SC-Exos) are advantageous for maintaining bone homeostasis. Mechanistic studies have shown that SC-Exos carry let-7c-5p and deploy it to regulate the TGF-β signaling pathway and promote the osteogenic differentiation of BMSCs, and SC-Exos can also promote the migration and angiogenesis of endothelial progenitor cells [177]. Another study of SC-Exos supported the notion that they can help BMSCs differentiate into osteoblasts by upregulating the TGFβ-1/SMAD2/3 signaling pathway and inducing macrophage M2 polarization [178]. Based on the above research, some researchers combined SC-Exos with porous Ti6Al4V scaffolds as a strategy for the treatment of bone defects [179]. Another group of researchers combined SC-Exos with bionic periosteum and found that it can target the damaged nerves of the periosteum after bone defects, thereby promoting angiogenesis and bone regeneration, and that the JNK3/MAPK pathway is crucial in this process [180].

Past confusion about the specific mechanism by which the nervous system affects bone regeneration has been resolved [176], and the connection between nerves and bone has become a current hot topic. The positive effects of nerve-derived exosomes on bone homeostasis have been confirmed. Numerous researchers have noted the potential of nerve-derived exosomes for clinical applications, but most of the current research in this field is focused on explaining specific clinical phenomena, few researchers have studied how nerve-derived exosomes affect bone homeostasis from a broader perspective. There is still much to learn and study about the specific mechanisms of exosomes and how they interact with biomaterials.

### Effects on bone by muscle-derived exosomes

Muscles and bones have a close relationship in terms of both anatomy and function, sarcopenia and osteoporosis often occur in the same individual [181, 182]. Various substances released by bone and muscle cells play a vital role in the interactions between bone and muscle, and existing studies have revealed that a variety of substances released by muscle and muscle stem cells contribute to fracture healing [183, 184]. In 2010, Guescini et al. first reported the production of exosomes by C2C12 myoblasts [185], and subsequent studies demonstrated that exosomes derived from C2C12 myoblasts can activate the β-catenin pathway through miR-27a-3p, thereby promoting osteogenic differentiation of MC3T3-E1 cells [186]. A recent study showed that Prrx2 carried in exosomes derived from C2C12 myoblasts can promote the transcriptional activation of MIR22HG, thereby activating the YAP pathway by sponging miR-128 and ultimately promoting the osteogenic differentiation of BMSCs [187].

Muscle exosome synthesis and function fluctuate depending on the body’s physiological condition; for instance, following exercise, muscle exosome production rises, and the majority of these exosomes are beneficial to bone metabolism [188, 189]. In contrast, the expression of miR-34a in muscle-derived exosomes increases significantly as people age, and miR-34a promotes skeletal muscle aging [190]. Interaction with BMSCs results in BMSC senescence and impedes osteogenesis [191]. These phenomena explain some of the beneficial effects of exercise on the body and the development of musculoskeletal disorders during aging.

Researchers have a new perspective on how muscle and bone interact, and exosomes play a role in the cellular communications between muscle and bone cells. This new perspective has helped researchers explain numerous previously misunderstood physiological or pathological processes. In the aging process, bones and muscles often degenerate together, and exosomes may be crucial in these processes, they mediate the mutual influence of bones and muscles during the aging process, so that several degenerative diseases often occur simultaneously in one person. Research into this phenomenon may offer new therapeutic options for several degenerative disorders that develop with aging.

### Effects on bone by blood-derived exosomes

Exosomes from diverse sources can travel through plasma to different organs and tissues throughout the body, leading to an abundance of exosomes from various tissues and organs in the blood. Numerous studies have demonstrated the potential for blood-derived exosomes in the early identification, diagnosis, and treatment of disorders associated with the skeleton [192–194]. Physiologically, the effects of blood-derived exosomes are affected by various factors, such as age and external stimuli. For example, an animal experiment showed that blood-derived exosomes from young mice had a higher concentration of miRNA-19b-3p than those from older mice, which can promote the osteogenic differentiation of BMSCs in aged osteoporotic mice, indicating the effect of age on blood-derived exosomes [195]. Other external factors, such as radiation exposure, can affect bone metabolism by changing the expression levels of miRNAs in blood-derived exosomes [196]. In addition, blood-derived exosomes have completely different effects in the bodies of healthy aged individuals versus those with osteoporosis or osteopenia [192].

These studies have shown that the role of blood-derived exosomes in the body is affected by various factors, and that clinicians may be able to assist in the diagnoses and treatments of some diseases by adjusting these factors. Researchers have found that miR-642a-3p [197], some lncRNAs [198] and surface protein markers (PCBP2, AARS, PSMB9 and VSIR) [199] in plasma-derived exosomes can be used as markers to help predict and diagnose osteoporosis for the early detection of the disease. Another study showed that the level of circulating exosomes in patients with femoral head necrosis was significantly lower than that in the control group, which shows that blood-derived exosomes can be used as diagnostic markers for some bone-related diseases [200].

There also have some researchers have focused their studies on the specific mechanisms of blood-derived exosomes acting in the human body. Platelet-rich plasma-derived exosomes (PRP-Exos) not only promote angiogenesis by activating the Akt and Erk signaling pathways but also prevent apoptosis in GC-induced necrosis of the femoral head by activating the Akt/Bad/Bcl-2 signaling pathway [201]. Moreover PRP-Exos also inhibit the M1 polarization of macrophages by regulating the MAPK and NF-κB pathways and regulating the phosphorylation of STAT6 to promote the M2 polarization of macrophages, thereby affecting bone metabolism. Moreover, previous studies have also demonstrated that PRP-Exos can reduce the expression of inflammatory mediators and apoptosis factors in vivo and can promote the autophagic degradation of NLRP3 by increasing the ubiquitination level of NLRP3 and reducing the production levels of IL-1β and Caspase-1 [202], thereby alleviating intervertebral disc degeneration. Serum-derived exosomes (serum-Exos) have been shown to modulate macrophage inflammation during bone defect repair, thereby promoting the expression of VCAM1 in HUVECs, leading to enhanced angiogenesis of HUVECs and ultimately promoting osteogenesis [203].

Under normal circumstances, blood-derived exosomes can promote osteogenesis through various pathways in the body, but their roles are easily affected by physiological conditions of the body or external factors, which leads to instability of their functions. But this special feature allows it to assist us in monitoring and diagnosing the occurrence of certain diseases to a certain extent. Blood-derived exosomes have good application prospects in the early detection and diagnosis of skeletal-related diseases.

### Effects on bone by exosomes from other sources

By extracting exosomes from human amniotic membrane extract (AME) and placental chorionic membrane extract (CME) for control experiments, Go et al. demonstrated that CME-derived exosomes can enhance the activity of ALP in osteoblast-like cells and promote the mineralization and osteogenic differentiation of osteoblast-like cells in a dose-dependent manner [204]. Wei et al. studied exosomes derived from stem cells from human exfoliated deciduous teeth (SHED-Exos) and found that SHED-Exos enhance osteogenesis by upregulating the expression of Runx2 and p-Smad5 while downregulating the expression of PPARγ, TNF-α, and IL-6 to suppress adipogenesis and inflammation [205]. Sun et al. noted that exosomes derived from sinus mucosa-derived cells and periosteum-derived cells can enhance the proliferation, migration and osteogenic differentiation of BMSCs, thereby accelerating osteogenesis [206].

In addition, fibroblast-like synoviocyte (FLS)-derived exosomes (FLS-Exos), especially exosomes secreted by RA-FLSs (RA-FLS-Exos), have received attention from researchers and are believed to stimulate bone growth [207]. This osteogenic effect is related to miR-486-5p contained in exosomes; miR-486-5p can promote osteogenic differentiation by reducing the expression of Tob1 and activating the BMP/Smad signaling pathway [208]. Studies have also shown that *Akkermansia muciniphila* (Akk), which is prevalent in the intestinal microbiota of children (CGM), can correct ovariectomy (OVX)-induced imbalance of bone metabolism and prevent osteoporosis. Further mechanistic studies have shown that the bone-protective effect mainly comes from EVs released by Akk, and that the introduction of EVs into bone tissue can reduce OVX-induced osteoporosis by enhancing osteogenic activity and inhibiting osteoclast formation [209].

Overall, these non-bone-derived exosomes have received extensive attention, and they affect bone homeostasis and the occurrence of bone diseases in various ways in vivo. An increasing number of researchers have begun to profoundly study the mechanisms of action of non-bone-derived exosomes in vivo and to attempt to apply them in clinical practice. For example, recent studies have shown that bone-targeted exosomes can be used to treat the treatment of bone loss in inflammatory bowel disease (IBD) [210]. Exosomes have promising application prospects in the treatment of bone diseases, and clinical trials on the safety and effectiveness of exosomes have been gradually carried out. We speculate that exosomes could be used as a good cell-free treatment for bone-related diseases in the future. However, even if clinical trials of exosomes achieve satisfactory results, there are still some problems that limit their clinical application that need to be solved, such as a lack of uniform production standards and efficient extraction technology, before they can be put into clinical use.

## Clinical application of exosomes

**Fig. 3:**
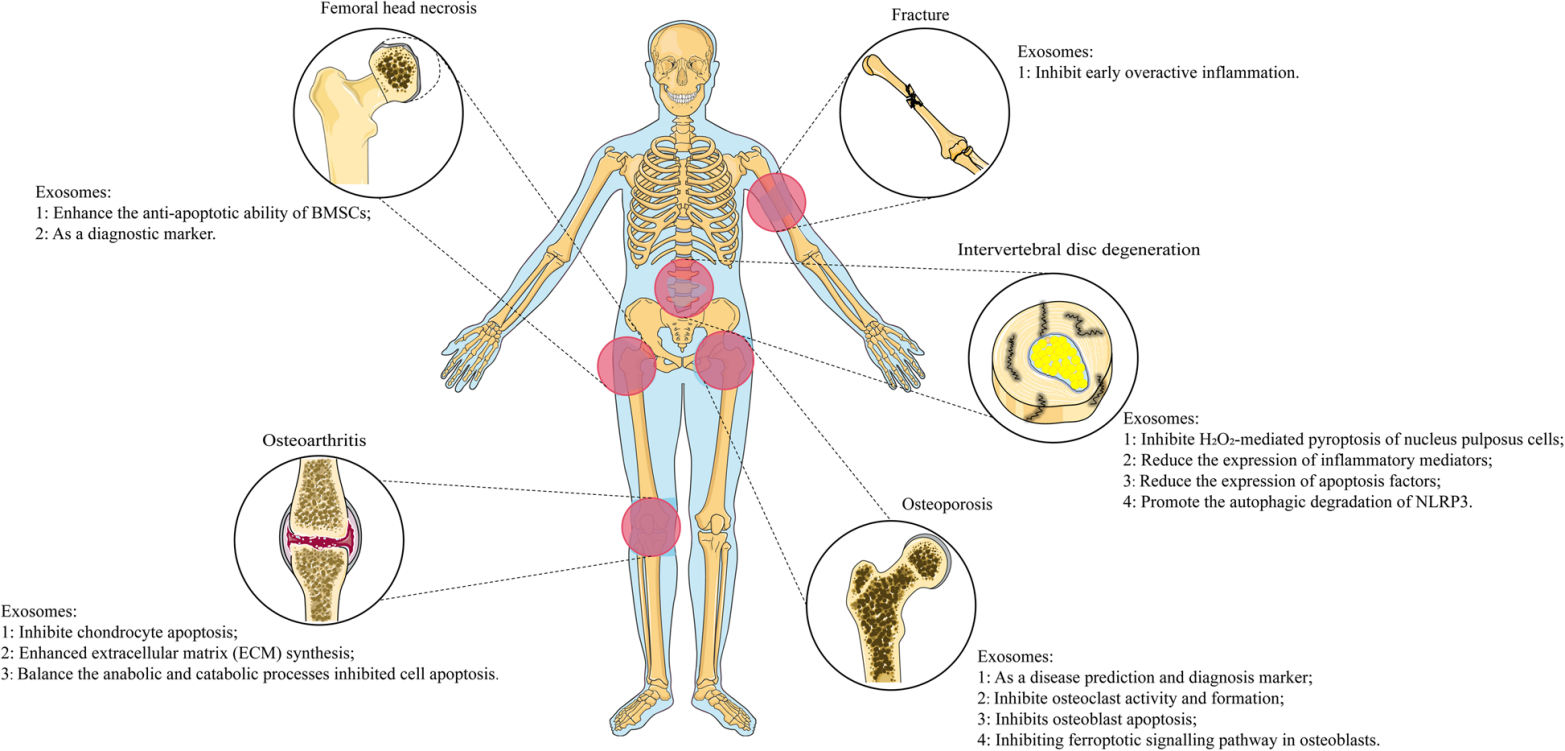
In most bone-related diseases, exosomes can promote the osteogenic differentiation and proliferation of BMSCs, inhibit the fat differentiation of BMSCs, promote the proliferation of osteoblasts and promote angiogenesis. In each disease, non-bone-derived exosomes have their own relatively unique functions

The diagnostic and therapeutic significance of exosomes in skeletal diseases has been shown in vitro and in animal experiments, and their mechanisms of action in a variety of bone-related diseases have been extensively studied (Fig. 3), but relevant clinical trials are still lacking. ClinicalTrials is a clinical trial database operated by the National Library of Medicine (NLM) under the National Institutes of Health (NIH) and the US FDA and is the largest existing clinical trial registry in the world. We searched it using the keyword “exosome” and found a total of 161 related clinical trials; the earliest one started in January 2014. These exosome-related clinical trials involve a variety of clinical diseases across eight major systems and cover multiple processes from the early diagnoses of diseases to prognostic treatment. Among these, there were 6 main projects studies to bones. Among these 6 studies, 1 study was in the “Active, not recruiting” phase, 1 study was in the “Recruiting” phase, 1 study was in the “Not yet recruiting” phase, and another 3 studies were “Completed”.

The study in the “Active, not recruiting” phase is dedicated to exploring whether the RNA profiles of circulating exosomes can be used as biomarkers for primary high-grade osteosarcoma lung metastasis, and the researchers aim to find lung metastasis and predict oncological outcome earlier through RNA analysis. The study in the “recruiting” stage is a phase II clinical trial regarding the clinical efficacy of exosomes in degenerative meniscus injury. The researchers aim to compare synovium-derived mesenchymal stem cells (SF-MSCs) to exosomes secreted by synovial-derived mesenchymal stem cells (SF-MSC-EXs) in the treatment of degenerative meniscus injury to determine the clinical effect of exosomes and to propose a new treatment scheme for degenerative meniscus injury. In addition, they aim to guide the use of MSC exosomes in other tissue and cell damage pathologies, immune pathologies or chronic inflammatory diseases. The clinical trial in the “Not yet recruiting” stage is a phase I clinical trial, the goal of which is to inject exosomes derived from mesenchymal stem cells (MSC-Exos) into the joints of patients with knee osteoarthritis to determine the therapeutic efficacy and safety of this treatment.

Finally, the other three clinical trials have been declared “Completed.” One used exosome microfluidic chips to establish a combination of exosome subgroup level markers (exosome barcodes) for the early diagnosis of osteosarcoma recurrence in the lung and aimed to use this as a basis for monitoring the early responses to second-line treatments of recurrent osteosarcoma. This study was announced to have been completed on September 30, 2022. The second completed study injected exosome-rich platelet-rich plasma (PRP) into intervertebral discs to study whether it can improve low back pain caused by intervertebral disc disease. This study was announced to have been completed on July 25, 2022. The final completed project aimed to identify a set of significantly dysregulated miRNAs in circulating exosomes of patients with bone metastases through appropriate molecular screening methods and to determine the biological mechanisms by which they function during bone metastases. A final aim was to obtain a panel of biomarkers that can predict cancer bone metastasis. This study was declared complete on December 31, 2021. Unfortunately, to date, none of the three completed clinical trials above have submitted their results to the ClinicalTrials database.

In summary, exosome-related clinical trials have steadily come to the attention of researchers over the past decade. Although there have not been many large-scale trials in the field of orthopedics, and the majority of those studies have not yet produced results, the publication of these clinical trial results and further exploration of the clinical uses of exosomes can be anticipated based on the positive results obtained in previous animal experiments and mechanistic studies. However, even if ideal results are obtained in clinical trials, there are still many issues for researchers to explore and solve in the clinical application of exosomes. Even if we put aside the standardization and optimization steps involved in exosome preparation, there are still issues to be resolved, such as how to collaborate with bioengineered scaffolds to increase exosome efficacy, how to individually modify the therapeutic dose throughout the course of treatment, and how to best coordinate the use of exogenous exosomes with the body’s endogenous exosome secretion. It is imperative to investigate these issues further prior to the official use of exosomes in clinical settings.

## Summary and outlook

Since exosomes were first discovered in 1983, they have received extensive attention from researchers [16]. As a class of extracellular vesicles with a size of 30–100 nm, exosomes can play important roles in metabolism, immune surveillance, angiogenesis, inflammatory response, and tumor development [7]. Exosomes can be secreted by a variety of cells (including various stem cells, osteoclasts, osteoblasts, and endothelial cells, etc.). In this review, we systematically evaluated the existing studies on non-bone-derived exosomes and showed that most non-bone-derived exosomes have positive effects on bone in vivo. The results support the use of exosomes in the clinical treatment of bone-related diseases.

Nonbone-derived exosomes are linked to bone homeostasis and the mechanisms of bone-related illnesses; they can not only directly control the development of osteoblasts and osteoclasts but also affect bone metabolism by stimulating angiogenesis and other means. The specific mechanisms by which exosomes act on bone are closely related to the various substances carried by exosomes, especially RNA, showing the diversity of their functions in their interactions with target cells [24, 27–29]. This shows the ability of exosomes to modulate various functions of recipient cells, which shows great potential in the diagnosis and treatment of orthopedic diseases [211]. In recent years, exosome research has made substantial progress, having been greatly stimulated by the difficulties encountered during the treatment of bone-related diseases and the shortcomings of the practical application of stem cells in terms of immune rejection. Our review systematically summarizes the existing research results regarding non-bone-derived exosomes. This can help researchers better understand the mechanisms of action of non-bone-derived exosomes in vivo and provide a reliable theoretical basis for the clinical applications of exosomes. Under the guidance of these theories, and due to their characteristics of high stability, nonimmunogenicity, and strong targeting, exosomes may replace stem cell therapy in the future and become an effective tool for the treatment of bone-related diseases.

However, although exosomes have good clinical prospects, as a relatively new technology in the field of tissue engineering, there are still many problems to be solved before they can be put into clinical application. To date, some technical, functional and safety issues of exosome-based pharmaceutical preparations are still unsolved, and there are no clear standards of clinical parameters in the production process. The source and quality of exosome samples have great impacts on the accuracy and reliability of research, but there are no standardized collection and processing methods, and the purity and consistency of samples are therefore difficult to guarantee [15]. In addition, exosome identification and quantification methods are not standardized or sufficiently uniform, and the methods and techniques used in different studies vary, thus, more accurate and reliable methodologies that can help researchers more accurately determine the molecular composition of exosomes need to be developed. Most existing exosome research is focused on cell and animal models, and specific clinical applications are still largely unknown. Whether the tail vein injection method commonly used in exosome experiments can accurately deliver exosomes to the site of disease also requires further research.

Due to the limitations of the production process, the currently used exosome extraction methods are not sufficient. The extraction efficiency of exosomes is low, which makes it impossible to use them in clinical practice on a large scale and greatly limits their clinical application prospects [212, 213]. Moreover, the genetic information contained in exosomes is still not fully elucidated. Although researchers have clearly analyzed the composition of exosomes, due to the complexity of the molecules carried by exosomes, the content and function of exosomes from different sources vary greatly. Few researchers have been able to adequately analyze and utilize all the characteristics of exosomes, and most researchers only focus on the role of monofunctional molecules carried by exosomes in vivo. Finally, before introducing exosomes into the clinic, we need to study their distribution in vivo after injection to check whether exosomes accumulate in nontarget organs and cause unintended side effects in these organs. There is still a long way to go for exosomes to be widely used clinically.

In short, although exosomes have good clinical prospects, there are still many problems to solve. Future researchers should start with how to make full use of all the characteristics of exosomes, and at the same time they should improve the extraction efficiency of exosomes to increase productivity and strive to solve the safety problems of exosomes as pharmaceutical preparations. We have reason to believe that with the development of biotechnology, after each of these problems is solved, the role of non-bone-derived exosomes in the maintenance of bone homeostasis, as well as the clinical diagnosis, monitoring and treatment of bone-related diseases, will be fully elucidated.

## Conclusion

In the past decade, research on the regulatory effect of non-bone-derived exosomes on bone has made significant progress. Its regulatory effect on bone and the underlying mechanisms have been extensively studied. However, many problems with non-bone-derived exosomes have not been resolved. Moreover, future clinical trials are needed to show whether they can be used clinically, and we need to further compare their advantages and disadvantages with stem cell therapy. Although much remains to be done before non-bone-derived exosomes can be used clinically, their role in bone homeostasis provides us with a new perspective for the treatment of bone diseases.

## Data Availability

Not applicable.

## Abbreviations

AD-Exos: Adipose-derived exosomes
ALP: Alkaline phosphatase
AMSC-Exos: Exosomes derived from adipose tissue-derived mesenchymal stem cells
Bax: B-cell lymphoma-2 associated X
BMDMs: Bone Marrow-Derived Macrophages
Bcl-2: B-cell lymphoma-2
BMP-2: Bone morphogenetic protein 2
BMSCs: Bone Marrow Mesenchymal Stem Cells
CREKA: Cysteine–arginine–glutamic acid–lysine–alanine
Coralline hydroxyapatite: CHA
DF-PEG: Difunctionalized polyethylene glycol
DUSP1: Recombinant Dual Specificity Phosphatase 1
ECM: Extracellular Matrix
EC-Exos: Endothelial cell-derived exosomes
EPC-Exos: Exosomes derived from endothelial progenitor cells
Erk: Extracellular regulated protein kinases
ESCRT: Exosomal-sorting complex that is required for transport
EVs: Extracellular Vesicles
GCS: Glycol chitosan
GelMA: Gelatin methacrylate
GIONFH: Glucocorticoid-induced osteonecrosis of the femoral head
GS-PDA-Exos: Gelatin sponge/polydopamine scaffold
HA: Hyaluronic acid
HIF-1α: Hypoxia inducible factor-1α
HPDLSCs: Human periodontal ligament stem cells
HUVECs: Human umbilical vein endothelial cells
H/SD: Hypoxia and serum deprivation
IMC: Intrafibrillar mineralized collagen
Intraluminal vesicles: ILVs
IPFP: Infrapatellar fat pad
IPN: Interpenetrating polymer network
IRS-1: Insulin receptor substrate 1
IVDD: Intervertebral disc degeneration
JNK: c-Jun N-terminal kinase
Klf4: Krüppel-like factor 4
MAPK: Mitogen-activated protein kinase
MIF: Migration inhibitory factor
MVBs: Multivesicular bodies
M1-Exos: M1 macrophage-derived exosomes
M2-Exos: M2 macrophage-derived exosomes
nSMase2: neutral sphingomyelinase 2
NLRP3: NOD-like receptor thermal protein domain associated protein 3
Nrf2: NF-E2-related factor 2
OCN: Osteocalcin
OA: Osteoarthritis
OVX: Ovariectomized
PA: Phosphatidic acid
PC: Phosphatidylcholine
PCNA: Proliferating cell nuclear antigen
PD-L1: Programmed death ligand 1
PE: Phosphatidyl-ethanolamine
PI: Phosphatidylinositol
PI3K: Phosphoinositide 3 kinase
PLGA: poly(lactic-co-glycolic acid)
PPARγ: Peroxisome proliferator-activated receptor γ
PS: Phosphatidylserine
RANK: Receptor Activator of Nuclear Factor-κ B
RANKL: Receptor Activator of Nuclear Factor-κ B Ligand
Ras: Renin-angiotensin system
RUNX2: Runt-related Transcription Factor 2
SANFH: Steroid-induced avascular necrosis of the femoral head
SDF-1α: Stromal cell-derived factor-1α
SF: Silk fibroin
SIK2: Salt-inducible kinase 2
SIK3: Salt-inducible kinase 3
SM: Sphingomyelin
Smad1: Mothers against decapentaplegic homolog 1
TBI: Traumatic brain injury
TNF-α: Tumor necrosis factor-α
ucMSC-Exos: Exosomes derived from umbilical cord-derived mesenchymal stem cells
VEC: Vascular endothelial cell
VEGF: Vasoactive Endothelial Growth Factor
β-TCP: β-tricalcium phosphate
